# Computer-assisted and image-guided surgery for surgical lymph-nodal staging of gynecologic cancer in the era of digital and robotic surgery: a review of current evidence

**DOI:** 10.1016/j.eclinm.2026.103869

**Published:** 2026-06-15

**Authors:** Matteo Pavone, Lise Lecointre, Nicolò Bizzarri, Anna Fagotti, Denis Querleu, Barbara Seeliger

**Affiliations:** a Institute of Image-Guided Surgery, IHU Strasbourg, Strasbourg, France; b Research Institute Against Digestive Cancer, IRCAD, Strasbourg, France; c UOC Ginecologia Oncologica, Fondazione Policlinico Universitario A. Gemelli, IRCCS, Catholic University of the Sacred Heart, Rome, Italy; d ICube, UMR 7357, CNRS, INSERM U1328 RODIN, University of Strasbourg, Strasbourg, France; e Department of Gynecologic Surgery, University Hospitals of Strasbourg, Strasbourg, France; f Department of Digestive and Endocrine Surgery, University Hospitals of Strasbourg, Strasbourg, France

**Keywords:** Computer-assisted image analyses, Digital surgery, Robotic-assisted surgery, Artificial intelligence, Computer vision, Image-guided surgery, Optical biopsy, FF-OCT, High-frequency ultrasound

## Abstract

Accurate lymph node staging is central to prognostic stratification and therapeutic decision-making in gynecologic oncology. Yet, conventional systematic lymphadenectomy frequently results in excessive treatment for node-negative patients, who represent the majority of cases, and is associated with significant morbidity. This review highlights how digital and imaging technologies are redefining intraoperative lymph node assessment. Real-time optical biopsies with full-field optical coherence tomography or high-frequency ultrasound, coupled with artificial intelligence–assisted image analysis, promise to enhance the precision, efficiency, and safety of nodal evaluation. Although the impact of robotic-assisted laparoscopy on oncologic outcomes compared with conventional approaches remains debated, its role as a versatile platform for the integration of digital assistance tools is steadily expanding. The convergence of these innovations is driving a shift from extensive dissections to selective, image-guided interventions to detect micrometastases intraoperatively. Ultimately, advances in intraoperative imaging and AI may make nodal excision unnecessary when a reliable negative assessment can be achieved. This paves the way for smart operating rooms where technology and surgical expertise converge to deliver safer, more efficient, and personalized oncologic care.

**Funding:**

This work was supported by French state funds managed by the ANR within the ‘Programme d'investissements d'avenir’ France 2030 (reference ANR-10-IAHU-02).

## Introduction

The assessment of lymph nodal status is of crucial importance in gynecological malignancies (ovarian, endometrial, cervical and vulvar cancers).^1^, ^2^, ^3^, ^4^, ^5^, ^6^ Lymph node involvement is a major component of the majority of prognostic classifications of gynecological malignancies, such as the FIGO classification, and is taken into account in all guidelines currently used in their management. The rate of positive lymph nodes in apparent early-stage cancers is far from low (14.2% ovarian, 10% endometrial, 15% cervical, and 10% of vulvar cancers).^7^, ^8^, ^9^, ^10^ Surgical strategy, prognosis and adjuvant treatment regimens are strongly related to nodal involvement.^11^ Frequently, systematic lymphadenectomies are performed for staging, diagnosis of skip metastases and to define the radiation field when adjuvant radiotherapy treatments are required.^1^, ^2^, ^3^, ^4^, ^5^ Yet, pelvic and/or para-aortic lymphadenectomy carries a risk of significant short- and long-term complications.^9^^,^^12^^,^^13^ In endometrial cancer, the recurrence-free survival rate drops from 87% in women without lymph node involvement to 71% and 36% with pelvic and aortic node involvement, respectively.^14^ Similarly, in cervical cancer, lymph node invasion is a critical prognostic factor, as reflected in the latest FIGO classification.^15^ Lymphatic spread is also a characteristic feature of epithelial ovarian cancer (EOC), even in apparent early stages.^16^ Studies assessing nodal involvement by performing systematic lymphadenectomy in all EOC stages have reported rates as high as 55% for pelvic and para-aortic nodal metastases in stages III and IV.^17^ However, the burden of surgical morbidity is even more difficult to bear in the majority of cases, regardless of the type of pelvic malignancy, in whom the lymph nodes are free of metastases.^18^^,^^19^ In other words, the majority of patients undergo an unnecessary, risky, and burdensome procedure that has no proven (or debated) impact on their survival rate. To solve this issue in early-stage cancers, sentinel lymph node (SLN) biopsy with ultrastaging analysis has become important, as it protects node-negative patients from the full lymphadenectomy-associated morbidity.^20^, ^21^, ^22^, ^23^ With the intention not to miss, for staging and therapeutic reasons, a positive node, gynecologic oncologists have performed extensive lymph node dissections, resulting in intraoperative and long-term complications impacting quality of life, specifically leg lymphedema. The increasing use of sentinel node only as a substitute to comprehensive lymphadenectomy is in line with the converging current concepts of de-escalation of surgery with the objective of reducing complications and of targeted lymph node staging made possible by the sentinel node biopsy. The accuracy of negative sentinel node as a predictor of the absence of positive lymph nodes has been demonstrated in vulvar and cervical cancer. In endometrial cancer, sentinel node procedure is recognized as a clinically meaningful compromise between omitting or performed comprehensive lymph node dissection, a long-lasting debate. Frozen section analysis of the sentinel lymph node is supported in cervical cancer to guide intraoperative decision-making, despite its time-consuming nature and limited sensitivity for detecting micrometastases, which is reportedly 43% lower than with ultrastaging.^3^^,^^24^ An alternative is to use SLN as a staging procedure only, possibly in conjunction with conization for cervix cancer, for definitive histopathology. Furthermore, reports of inadvertent failure to remove nodal tissue (“empty packets”: retrieval of fatty tissue without lymph nodes) in 2–20% of patients depending on their body mass index and surgeons’ experience, highlight the risks for understaging or overtreatment to be on the safe side.^25^^,^^26^ Additionally, SLN mapping can sometimes fail, making the SLN technique unusable and the patient under-staged, forcing surgeons to extend the lymphadenectomy. Even though efforts are focused on tailoring lymph node removal to avoid complications of extensive lymphadenectomies, the detection of pathological lymph nodes with micro- or macrometastases remains a challenge for pre- and intra-operative techniques.^27^

**Fig. 1:**
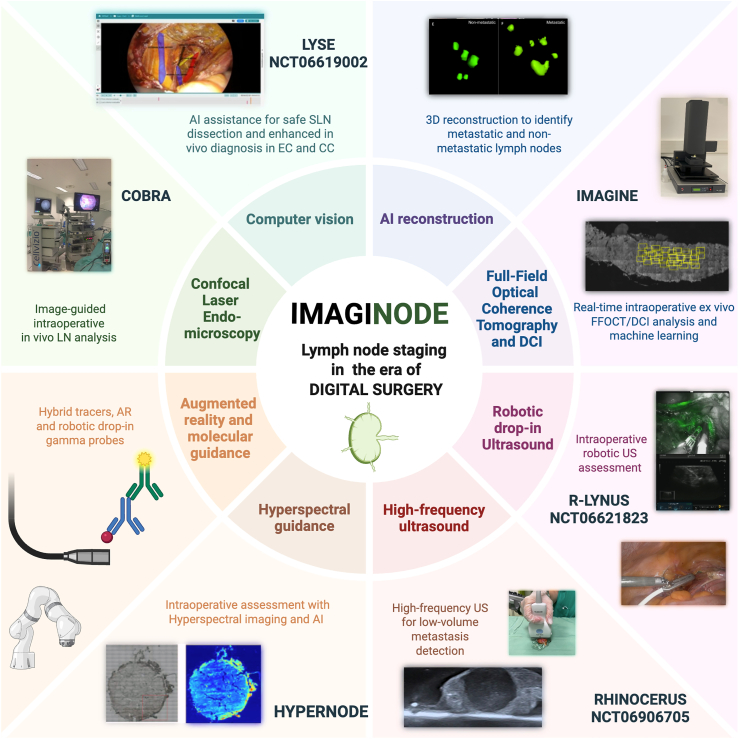
Surgical lymphnodal staging of gynecologic malignancies in the era of digital and robotic surgery. The most recent technological advances and their evaluation in ongoing trials are illustrated here, listing the various complementary novel methodologies in the inner circle, and corresponding study details in the outer fields. The main principles across these advances are improved localization of target nodes, and their intraoperative assessment in real time to tailor the extent of dissection. Localization can be improved by augmented reality, molecular-guided surgery, and intraoperative ultrasound assessment. It can further be enhanced by AI reconstruction, based on the automatization of 3D reconstructions of the relevant lymph nodes in imaging studies to differentiate between metastatic and non-metastatic ones,^28^ and by computer vision based on the automatic recognition of SLN in laparoscopic videos. Real-time optical biopsies with full-field optical coherence tomography, confocal laser endomicroscopy, hyperspectral laparoscopy or high-frequency ultrasound, in conjunction with artificial intelligence–assisted image analysis, could further enhance precision, efficiency, and safety of nodal assessment. AI = Artificial Intelligence, AR = Augmented Reality, CC = Cervical Cancer, EC = Endometrial Cancer, DCI = Dynamic Cell Imaging, FFOCT = Full-Field Optical Coherence Tomography, SLN = sentinel lymph node, US = Ultrasound. Figure created in https://BioRender.com, 3D reconstruction to identify metastatic and non-metastatic lymph nodes from^28^ published under CC BY 4.0.

In the era of digital surgery, emerging technologies can help overcoming current limitations. The implementation of image-guided and computer-assisted surgery with digital systems including machine learning technology embedded in robotic platforms is aimed at seamlessly integrating groundbreaking innovations into the operating room (Fig. 1). In the near future, these advances could enable a more personalized, technology-assisted surgical approach.^29^^,^^30^ The present review article provides an overview of currently and soon-to-be available technologies for intraoperative lymph node staging in gynecologic oncology surgery.

## Search strategy

We conducted a literature search to identify relevant studies evaluating the use of digital, computer-assisted, and image-guided technologies in surgical lymph node staging for gynecologic malignancies. Data for this review were identified by searches of PubMed/MEDLINE, SCOPUS, Google Scholar, and ClinicalTrials.gov databases. The following key terms and combinations thereof were used: *“sentinel node”*, *“lymph node”*, *“gynecology”*, *“digital surgery”*, *“image-guided surgery”*, *“artificial intelligence”*, and *“robotic surgery”*. Boolean operators and truncations were applied where appropriate.

The search was limited to articles published in English between January 1, 2015, and March 31, 2025, to include the latest evidence on technological advances. Additional references were identified by manually screening bibliographies of key articles. Both peer-reviewed original research and review articles were included. Conference abstracts and proceedings were only considered if they were directly related to previously published peer-reviewed studies, particularly for emerging technologies and clinical trial updates.

Eligible studies addressed one or more of the following domains: sentinel lymph node mapping, intraoperative lymph node assessment, image-guided and ultrasound-guided surgery, optical biopsy techniques, high-frequency ultrasound, artificial intelligence and computer vision for lymph node staging, and robotic-assisted lymphadenectomy in the field of gynecologic oncology.

## Current state of the art of lymph node staging

Sentinel lymph node biopsy represents the biopsy of the first node(s) draining the primary tumor. It has progressively replaced lymphadenectomy in gynecologic cancers to detect micro- and macro-metastases not detectable on preoperative imaging.^31^ This approach led to a ∼30% reduction in short- and long-term complications, while improving the detection accuracy of micrometastases^32^ — even when combined with lymphadenectomy.^9^^,^^25^^,^^26^^,^^33^^,^^34^ In specific scenarios, such as cervical cancer, surgical strategy is directly dependent on nodal status.^24^^,^^35^, ^36^, ^37^ Intraoperative identification of lymph node involvement can significantly alter the course of treatment, shifting from radical surgery to exclusive chemoradiation—thereby avoiding completion of hysterectomy.^38^ However, intraoperative frozen section assessment has limitations that undermine its reliability in guiding surgical decision-making. These include: I) a limited diagnostic accuracy (∼65%) compared to ultrastaging (72% when excluding isolated tumor cells); II) prolonged operative time, which increases healthcare costs and extends patient exposure to anesthesia; III) partial (frozen section) or complete (e.g., OSNA) tissue destruction, which limits definitive histopathologic evaluation.^18^^,^^37^^,^^39^ Table 1 provides an overview of clinical trials on lymph node staging, and Table 2 an overview of clinical trials on innovations in lymph node staging for gynecological cancers.

**Table 1:** Clinical trials on lymph node staging for gynecological cancers.

Acronym	Cancer type	Mapping/intervention vs comparator	Primary endpoint	Key results	Publication
SENTICOL I trial	Early cervical	SLN mapping (radiocolloid + blue dye) with completion PLND	NPV and sensitivity of bilateral negative SLNs	Bilateral negative SLNs accurately predict node-negative status	J Clin Oncol 2011
SENTIX trial	Early cervical	SLN biopsy with ultrastaging without systematic PLND	Safety/oncologic outcomes; mapping performance; intra-op pathology performance	LN biopsy without systematic PLND did not increase the risk of recurrence in patients with early-stage cervical cancer	Nature 2025
SENTIREC	Cervical & endometrial	Adoption of SLN algorithms; adjunctive FDG-PET/CT sub-studies	Diagnostic accuracy (sensitivity/NPV); algorithm validation; imaging accuracy	High mapping accuracy when SLN algorithm strictly followed; algorithm work shows safe/accurate strategies	Gynecol Oncol 2021–2024, Cancers 2025
FILM	Cervical & endometrial	ICG NIR fluorescence vs isosulfan blue	Detection of SLNs (overall and bilateral)	ICG was superior to blue SLNs than blue.	Lancet Oncol 2018
FIRES	Endometrial	SLN mapping with cervical ICG → completion lymphadenectomy	Sensitivity & NPV of SLN vs reference LND	Supports SLN as safe alternative to systematic LND for staging.	Lancet Oncol 2017
ASTEC trial	Endometrial	Standard hysterectomy ± systematic pelvic lymphadenectomy	Overall survival; recurrence-free survival	No survival benefit from systematic pelvic lymphadenectomy	Lancet 2009
SELLY	Apparent early-stage epithelial ovarian	Ovarian SLN mapping (ICG ± 99mTc) with reference lymphadenectomy/ultrastaging	Detection rate, sensitivity, NPV	Detection feasible, but sensitivity did not meet prespecified target; ultrastaging identified ∼⅓ of node-positive cases otherwise missed.	Eur J Cancer 2024, ASCO 2023
GROSNAPET	Vulvar	SLN biopsy + pre-op 18 F-FDG PET/CT to expand SLN eligibility	Accuracy of combined approach for nodal staging	Explored PET/CT to better select true node-negative pts and extend SLN indication; showed feasibility and outlined selection criteria.	EJSO 2017

SLN = sentinel lymph node; PLND = pelvic lymph-node dissection; ICG = indocyanine green; NIR = near-infrared; NPV = negative predictive value; FDG-PET/CT = fluorodeoxyglucose positron-emission tomography/computed tomography.

**Table 2:** Clinical trials on innovations in lymph node staging for gynecological cancers.

Acronym	Cancer type	Technology	Primary endpoint	Key results	Registration/journal
LYNOP	Endometrial, cervical, ovarian and vulvar	FF-OCT	Assessing the sensitivity, specificity and accuracy of FF-OCT in detecting LN metastasis (macro and micro)	High accuracy (97.6%), sensitivity (92.3%), and specificity (98.2%) of FF-OCT compared to histology	Nature Sci Rep. 2025
LYSE	Endometrial and cervical	Computer vision	Developing a deep learning-based model to intraoperatively assess the critical views of safety in SLN dissection	RECRUITING	NCT06619002
R-LYNUS	Endometrial and cervical	Drop-in ultrasound probe (3–5 MHz)	Reporting the sensitivity of in vivo RIOUS in metastasis detection (macro, micro and ITCs) from unstained LN	RECRUITING	NCT06621823
RHINOCERUS	Endometrial, cervical, ovarian and vulvar	High-frequency ultrasound probe (33 MHz)	Reporting the sensitivity of ex vivo HFUS in metastasis detection (macro, micro and ITCs) from fresh, unstained LN	RECRUITING	NCT06906705
IMAGINE	Endometrial, cervical, ovarian and vulvar	FF-OCT and Dynamic Cell Imaging	Assessing the sensitivity, specificity and accuracy of ex vivo FF-OCT and DCI in detecting LN metastasis (macro, micro and ITCs) on fresh, unstained LN	NOT YET RECRUITING	N/A
HYPERNODE	Endometrial, cervical, ovarian and vulvar	Hyperspectral imaging	Assessing the sensitivity, specificity and accuracy of Hyperspectral imaging in detecting LN metastasis (macro, micro and ITCs)	NOT YET RECRUITING	N/A
COBRA	Endometrial, cervical, ovarian and vulvar	Probe-based confocal laser endomicroscopy	Assessing the sensitivity, specificity and accuracy of pCLE in detecting LN metastasis in vivo (macro, micro and ITCs)	NOT YET RECRUITING	N/A

LN = lymph nodes; SLN = sentinel lymph node; FF-OCT = Full-Field Optical coherence tomography; RIOUS = robotic intra-operative ultrasound; HFUS = High-Frequency Ultrasound; ITC = isolated tumor cells.

## Lymph node staging via image-guided surgery

### Tracer-guided staging

Molecular image-guided surgery represents an emerging frontier for more accurate lymph node staging and less invasive gynecologic oncologic procedures.^31^ The most established technique is sentinel lymph node biopsy, performed using radioactive tracers (such as [99mTc] Tc-nanocolloid), non-specific fluorescent agents (such as indocyanine green, ICG), or hybrid tracers combining both signals to identify SLN. Detailed mapping of lymphatic drainage by integrating these agents with preoperative imaging modalities (such as SPECT/CT or PET/CT) enables targeted surgical planning and reduces unnecessary dissection.^40^

For example, in vulvar cancer, the combination of SPECT/CT with hybrid tracers enhanced detection of sentinel nodes in atypical locations (e.g., paravesical or gluteal regions), thereby minimizing the need for extensive lymphadenectomy.^41^ In endometrial and cervical cancer, intraoperative use of ICG in the FILM trial demonstrated superior bilateral detection rates compared to traditional dyes.^42^ In endometrial cancer, the combination of ICG and radiolabeled nanocolloids showed near-perfect sensitivity and negative predictive value, including the ability to identify para-aortic nodes in intermediate-to high-risk patients.^31^ In early-stage ovarian cancer, pilot studies validated the feasibility of sentinel node mapping through injection of tracers into the ovarian ligaments, allowing visualization of pelvic and para-aortic nodes.^22^ Moreover, in cases of recurrent or non-palpable tumors, radio-guided occult lesion localization techniques are being explored to support more precise resections.^31^

Intraoperative detection of sentinel nodes can be achieved using conventional gamma probes or robotic-compatible drop-in probes. When combined with freehand SPECT, these tools enable real-time three-dimensional reconstruction of patient anatomy.^40^ The integration of augmented reality (AR) with laparoscopic or robotic views allows surgeons to overlay anatomical and molecular images directly onto the surgical field, potentially enhancing dissection accuracy and reducing procedural risks.^40^ The evolution toward these digital, personalized approaches holds great potential for improving oncologic and functional outcomes in patients with gynecologic malignancies.

### Ultrasound-guided staging

Intraoperative ultrasound (IOUS) is emerging as a valuable complementary tool for lymph node staging in gynecologic cancers, particularly in endometrial and cervical carcinomas, as well as in the assessment of nodal recurrences in ovarian cancer.^43^^,^^44^ During open, laparoscopic or robotic procedures, high- or ultra-high-frequency probes, including miniaturized *drop-in* probes, enable real-time direct evaluation of lymph nodes^45^ to assess nodal morphology, echogenicity, and vascular patterns, thereby supporting targeted selection of nodes for biopsy or removal.^46^^,^^47^

Preliminary studies have demonstrated that IOUS can help identify nodal features suggestive of metastasis, reducing the risk of understaging and potentially limiting the need even for SLN biopsy.^48^ The integration of ultrasound into robotic surgery with conventional laparoscopic probes or modern robot-compatible *drop-in* probes has seen wide adoption in other surgical fields, particularly hepatobiliary surgery.

Small-sized probes (2.5–5 cm) with a dorsal fin that can be easily manipulated by robotic instruments can be inserted through standard 10 mm accessory trocars, making IOUS scalable to all robotic platforms. The combination of robotic instrument articulation and the probe's flexible cable allows access to anatomical regions that are difficult to reach using standard laparoscopic ultrasound devices. Additionally, software interfaces such as TilePro (Intuitive Surgical, Sunnyvale, CA, USA) enable simultaneous visualization of ultrasound and robotic camera views, enhancing surgical precision and supporting more focused, conservative, and personalized procedures.^45^ In gynecologic oncology, the ongoing R-LYNUS trial (NCT06621823) aims to evaluate the sensitivity of robotic intraoperative ultrasound (RIOUS) in detecting macro-, micro-, and isolated tumor cell (ITC) metastases from fresh, unstained sentinel lymph nodes in patients with endometrial and cervical cancer.^49^

**Fig. 2:**
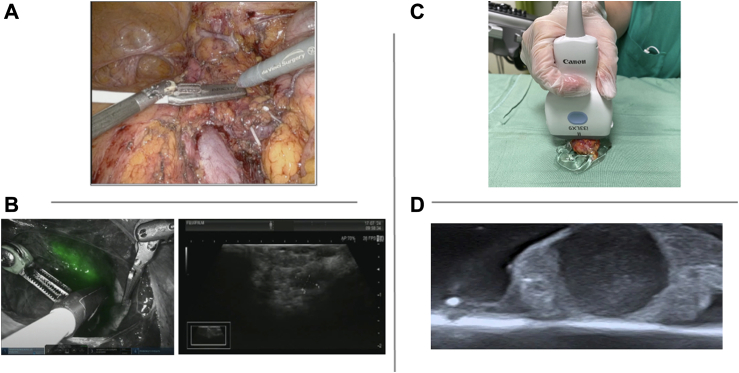
A-B) intraoperative robotic ultrasound with drop-in probes; A) drop-in probe, in vivo analysis of a sentinel lymph node B) near-infrared visualization with indocyanine green highlights and ultrasound images. C–D) High-frequency ultrasound C) ex vivo analysis of a pelvic lymph node; D) ultrasound appearance of a metastatic lymph node.

The role of RIOUS in reducing the risk of *empty packets*—i.e., retrieval of non-nodal tissue—is already apparent,^49^ but the frequency range of currently available *drop-in* probes (e.g., 2–12 MHz, Arietta L43K, Hitachi, Japan) is not sufficient to detect micrometastases (<2 mm). In this context, high-frequency ultrasound (HFUS) at frequencies between 22 MHz and over 70–100 MHz provides exceptional superficial spatial resolution, making it suitable for dynamic assessment of target lymph nodes.^50^ This improved resolution may facilitate the detection of subtle morphological alterations associated with micrometastases or early metastatic foci, thereby supporting more accurate intraoperative lymph node selection and guiding more refined surgical decision-making. In contrast to conventional ultrasound probes, HFUS offers a highly detailed visualization of nodal architecture, including the cortex, corticomedullary interface, and hilum.^51^ In gynecologic oncology, HFUS may be used ex vivo, e.g., on freshly excised lymph nodes in cervical cancer, or in vivo, e.g., on surgically accessible inguinal nodes in vulvar cancer.^52^ A prospective trial is ongoing (RHINOCERUS NCT06906705) to evaluate the accuracy of this technique in detecting lymph nodal involvement^52^ (Fig. 2).

### Intraoperative optical biopsy

**Fig. 3:**
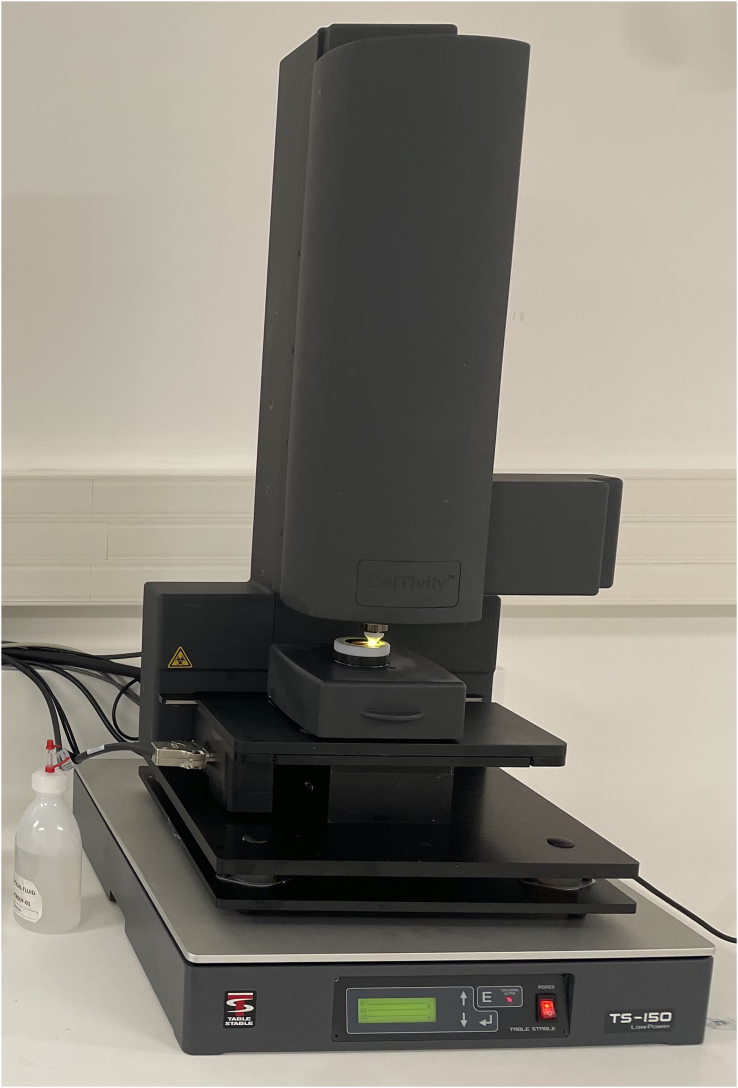
Full-Field Optical Coherence Tomography setup at IHU Strasbourg.

**Fig. 4:**
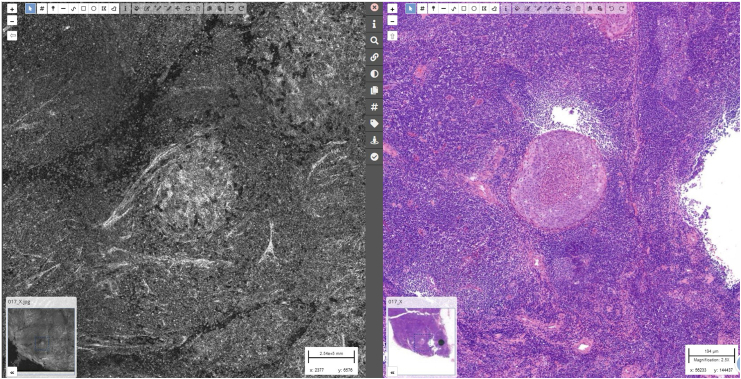
Positive lymph node with a micrometastasis (<2 mm) seen on Full-Field Optical Coherence Tomography and histology images. A low-resolution section of this image was published by the authors in^57^ under CC BY-NC-ND 4.0.

Intraoperative techniques to rapidly analyze lymph node tissue either in vivo or *ex vivo*—without the need for staining, or by using non-destructive contrast agents—show promise for real-time surgical decision-making support.^53^ Among these, Full-Field Optical Coherence Tomography (FF-OCT) stands out as a microscope-sized device suitable for a dedicated room in the OR suite for immediate ex vivo tissue analysis (Fig. 3). Utilizing incoherent illumination, FF-OCT produces histopathology-like images within 5–15 min according to the number of acquisitions in 2D or 3D, with micrometer-level resolution.^54^ Tissue is scanned at pre-selected depths, enabling a form of optical ultrastaging without staining or multiple cuts of the specimen.^54^ Dynamic Cell Imaging mode can be added, capturing cellular metabolic activity within slightly longer acquisition times of short videos per frame. The endogenous metabolic contrast distinguishes viable tumor tissue from reactive nodes, adding functional to morphological information.^55^ A pilot study by our group demonstrated concordance between FF-OCT and hematoxylin-eosin (H&E) imaging in the diagnosis of nodal metastasis from ovarian cancer,^56^ and the prospective clinical study evaluating the diagnostic performance of FF-OCT in detecting lymph node micro- and macrometastases showed an accuracy of 97.6%, sensitivity of 92.3%, and specificity of 98.2%^57^ (Fig. 4). Emerging optical technologies with potential for in vivo lymph node assessment include hyperspectral imaging (HSI) and confocal laser endomicroscopy. Both technologies, compatible with laparoscopic and open-surgical systems, provide advanced spectral and spatial analysis currently at an early stage of implementation.^58^^,^^59^ Confocal microscopy techniques—including CLE, pCLE, FCM, and RCM—enable real-time, high-resolution imaging: CLE with targeted fluorochromes may support intraoperative histomorphological assessment and pCLE has shown high concordance with immunohistochemistry in preclinical models, whereas FCM offers rapid ex vivo discrimination of lymph node status, and RCM holds potential for superficial nodal analysis.^60^

### Toward an integrated multimodal approach

Image-guided lymph node staging currently relies on a spectrum of complementary technologies that differ in their underlying physical principles, intraoperative applicability, and diagnostic depth. Molecular mapping with radiotracers or near-infrared fluorescence remains the most established technique, offering high sensitivity and panoramic visualization of lymphatic pathways. It enables precise localization of sentinel nodes, but it provides limited information on tissue composition and requires prior tracer injection.^31^^,^^61^

Intraoperative ultrasound adds real-time anatomical and vascular information, allowing direct visualization of nodal structures and their relationship with surrounding vessels. It facilitates selective dissection and helps avoid incomplete retrieval of nodal tissue. However, its diagnostic accuracy is still operator-dependent and constrained by acoustic access, particularly in deep pelvic or paraaortic basins. High-frequency ultrasound improves spatial resolution, potentially detecting microarchitectural alterations associated with micrometastases, although to date its use is only for superficial or ex vivo settings.^49^^,^^52^

Optical biopsy techniques, such as full-field optical coherence tomography and confocal laser endomicroscopy, provide near-histological visualization within seconds, offering functional and morphological assessment without tissue destruction. These modalities enable immediate verification of suspicious nodes or margins but are limited by small fields of view. Hyperspectral imaging, still at an early stage of clinical translation, provides wide-field, label-free assessment of tissue oxygenation and metabolic contrast, with promising applications for intraoperative discrimination between healthy and metastatic nodes, although real-time implementation and algorithmic standardization remain challenges.^57^^,^^58^^,^^60^

Rather than competing, these modalities are converging towards a multimodal paradigm in which molecular imaging identifies the drainage pathway, ultrasound refines anatomical localization, and optical or spectroscopic technologies provide microscopic confirmation. When integrated within digital or robotic platforms, these methods can be co-registered in real time—transforming image-guided lymph node staging from a series of isolated procedures into a unified digital workflow that combines mapping, guidance, and in situ diagnosis.

## Artificial intelligence-based lymph nodal staging

Artificial intelligence with machine learning and computer vision is an emerging tool to enhance lymph node staging in gynecologic malignancies.^28^ Recent advances in computer vision and deep learning are redefining how surgical safety and lymph node assessment can be evaluated in real time. The integration of AI into minimally invasive gynecologic oncology allows quantitative analysis of surgical videos, enabling the identification of anatomical landmarks, detection of critical intraoperative events, and assessment of adherence to standardized procedural steps. In this context, the multicentric LYSE study (NCT06619002) represents a pivotal initiative to implement video-based quality assessment during sentinel lymph node (SLN) dissection in uterine malignancies. The study aims to establish and automatically recognize the so-called Critical Views of Safety (CVS)—defined by the exposure of key anatomical structures such as the ureter, obliterated umbilical artery, and internal iliac vessels—before lymph node excision. These standardized spatial references, adapted from consensus-based surgical guidelines, form the visual foundation for AI training and validation.

By annotating laparoscopic and robotic videos according to the CVS criteria, the LYSE project seeks to develop deep learning models capable of automatically detecting surgical phases, identifying structures, quantifying ICG fluorescence intensity, and correlating these features with histopathologic nodal status. This video-based approach enables an objective assessment of surgical proficiency, reduces variability across centers, and supports the surgeon during the learning curve by providing context-aware visual feedback. Beyond safety monitoring, such algorithms may allow intraoperative prediction of metastatic involvement by linking fluorescence signal patterns with pathologic findings, paving the way for real-time digital pathology integration.^62^

In parallel, AR integrated into robotic surgery has demonstrated the ability to overlay manually segmented preoperative CT images onto the live endoscopic view.^28^^,^^63^ This fusion allows precise identification of target pelvic SLNs and surrounding vascular structures, achieving >90% overlay accuracy and <6% overflow rates between the segmented image and real anatomy. The combination of robot-assisted surgery and AR, as developed by our group,^64^ represents a major technological advance for the intraoperative detection of the pelvic sentinel lymph node (SLN) in endometrial cancer. The experimental system integrates a servo-controlled robotic arm, ensuring stability and precision of optical positioning, with an AR module enabling real-time fusion of segmented preoperative SPECT/CT volumes with the laparoscopic video stream. This dynamic overlay relies on an optimized registration chain that minimizes cumulative transformation errors and ensures reliable spatial concordance between 3D models and the actual surgical scene. The AR platform was specifically designed to handle pelvic anatomical deformations by integrating intrinsic registration algorithms based on vascular landmarks, allowing continuous recalibration throughout the surgical procedure. Furthermore, the system incorporates a user interface displaying both overlaid anatomical models and navigation cues, tailored to the ergonomics of laparoscopic and robotic workflows. Preclinical evaluations on porcine and human arterial models demonstrated excellent precision in SLN localization compared with conventional techniques. The integration of AR reduces the surgeon's cognitive load by providing direct and continuous visual guidance, while facilitating the identification of deep-seated structures or those adjacent to critical anatomical areas. Robotic assistance enhances surgical ergonomics, stabilizes the operative field, and contributes to procedural standardization, thereby potentially reducing false-negative rates and improving inter-operator reproducibility. This multimodal approach paves the way for safer, less invasive, and potentially more efficient nodal staging in complex pelvic oncology. Such technology offers a valuable tool to support both systematic lymphadenectomy and SLN biopsy in endometrial and cervical cancer cases.

One could extrapolate the data from breast cancer studies for potential improvement in gynecologic oncology. Predictive models based on 3D reconstruction of preoperative CT —including morphometric features such as lymph node sphericity—achieved higher diagnostic accuracy than conventional ultrasound or CT alone. Notably, the combined use of 3D models with ultrasound yielded a correct classification rate of 92.3%, underscoring the added value of integrating morphometric data with AI.^28^ These approaches hold significant translational potential across the field of gynecologic oncology, particularly for refining target node selection and avoiding unnecessarily extensive dissections.^28^

## Technological advances

When it comes to robot-assisted minimally invasive treatment of gynecological tumors, it is important to avoid jumping to conclusions. There is currently no evidence demonstrating superiority of robotic assistance over traditional laparoscopy for gynecologic oncology outcomes. Compared to laparotomy, robot-assisted laparoscopy offers significant perioperative advantages, including reduced blood loss, shorter hospital stays and fewer complications, but not when compared to standard laparoscopy. The only superiority randomized trial comparing the safety and clinical efficacy between robot-assisted and traditional laparoscopy for patients with gynecologic cancer requiring minimally invasive surgery (cervix, uterus or ovary, phase III, ROBO-GYN trial, NCT01247779) did not reveal superior results for robotic assistance in terms of severe perioperative morbidity, based on a patient cohort from 2010 to 2015 across French hospitals.^65^ Furthermore, the only randomized controlled trial comparing minimally invasive and open surgery outcomes in cervical cancer reported similar survival rates within the minimally invasive surgery group.^38^

In addition, four well-conducted meta-analyses addressing early-stage cervical cancer^66^, ^67^, ^68^ and endometrial and cervical cancer^69^ came to the same conclusion with no evidence of superiority of robotic assistance among minimally invasive surgery techniques. The popular claim that ‘robotic surgery is advantageous for complex surgical procedures in the deep and narrow pelvic cavity’ is thus not supported by any high-quality patient outcome data. Whether such advantages can be demonstrated in obese patients is currently under investigation in a multi-institutional phase III trial (endometrial cancer, BMI ≥30, RObese trial, NCT05974995).^70^ Nevertheless, it is worth noting that in other oncologic fields—such as rectal cancer—recent randomized controlled trials have demonstrated a potential benefit from the integration of robotic technologies to optimize surgical outcomes.^71^

Within the paradigm of precision medicine, the use of sophisticated and costly surgical technologies remains to be critically evaluated and clearly justified based on clinical necessity for its routine implementation. The use of fluorescence imaging remains essential for accurate SLN detection. The current era of surgical de-escalation aims at reduced morbidity while maintaining optimal oncological outcomes. As underlined by the SENTIX trial results,^37^ the adoption of sentinel lymph node biopsy alone for early-stage cervical cancer has significantly simplified surgical management, as opposed to systematic pelvic lymphadenectomy.

The evolution from open surgery to laparoscopy has marked a more transformative path than the addition of robotic assistance to minimally invasive surgical techniques. Robotic assistance systems available to date—despite their diversity—are largely in clinical use as modernized laparoscopic cameras and instruments. Are we only observing an increased use of an expensive tool with no demonstrated benefit, along with a loss of laparoscopic skills and training in academic institutions? Is it therefore time to give up on robotics for gynecologic cancer surgery in disillusionment? We don't think so, because the potential of the technology is far from being fully exploited.^72^ Trends in further developments are increasingly becoming apparent not only at engineering conferences, but also at surgical conferences. Robotic platforms serve as digital interfaces that enable real-time integration of further assistive technologies and data analysis. This era of digital surgery is set to incorporate evolving technologies such as intraoperative imaging, augmented reality, and artificial intelligence to provide patient-centered precision surgery (Fig. 1). As recently highlighted, the “real benefits” of robotic assistance in surgery may not yet lie in improved perioperative outcomes, which is still under debate, but rather in its capacity to integrate multimodal imaging and data streams, support surgical decision-making, and enable telementoring and remote surgical collaboration.^30^^,^^63^^,^^73^ The progressive integration of imaging tools within robotic platforms has opened the field to tele–image-guided surgery, where multiple surgeons can share control and imaging feedback in real time across different locations. In lymph node staging for gynecologic malignancies, this model allows remote collaboration with experts in nodal ultrasound or pelvic anatomy through multi-console systems equipped with in-house ultrasound interfaces. Such interaction supports accurate identification of sentinel and para-aortic nodes, validation of anatomical exposure, and safer dissection. Tele–image-guided surgery may therefore contribute to standardizing nodal assessment and extending specialized expertise to centers with limited resources.^30^

## Outstanding questions

Despite remarkable technological progress, several unresolved questions and limitations must be addressed before emerging digital and robotic solutions can be safely and effectively implemented for intraoperative lymph node staging in gynecologic oncology.

The sentinel lymph node procedure—although increasingly standardized—remains technically demanding and operator-dependent. Mapping accuracy and retrieval rates vary widely across centers, particularly in low-volume institutions or in obese patients, where the rate of “empty packets” or failed mapping may reach up to 20%.^26^ Its diagnostic performance therefore depends heavily on the surgeon's experience, the quality of preoperative imaging, and adherence to defined critical views of safety. Real-world data registries and structured training programs are essential to ensure that SLN biopsy achieves consistent and reproducible results beyond specialized centers.^62^

Large, multicenter randomized controlled trials are needed to definitively demonstrate non-inferiority of sentinel-node–only approaches compared with systematic lymphadenectomy, both in terms of oncologic safety and long-term survival.^37^ Moreover, these trials should assess whether the incorporation of digital tools—such as intraoperative ultrasound, optical biopsy, or augmented reality—can further enhance diagnostic accuracy or influence treatment algorithms in a clinically meaningful way. Pathological ultrastaging has expanded the detection spectrum of nodal disease by revealing low-volume metastases, particularly isolated tumor cells (ITCs). However, the biological and prognostic significance of these findings remains uncertain, and current guidelines provide no consensus on adjuvant management. The arbitrary cut-offs of 0.2 mm and 2 mm used to distinguish ITCs, micrometastases, and macrometastases fail to account for the heterogeneity of tumor biology.^74^ Molecular assays such as one-step nucleic acid amplification (OSNA) quantify cytokeratin copy numbers rather than lesion size, highlighting the need for a molecular rather than morphometric classification of nodal disease.^39^ Establishing clinical correlations between molecular burden and recurrence risk represents a major research priority. Technological barriers still hinder full clinical integration of these emerging tools. Optical imaging and high-frequency ultrasound are limited by penetration depth, restricting in vivo assessment to superficial or ex vivo settings. Real-time data fusion across modalities—combining molecular, ultrasound, and optical information—remains technically challenging, particularly in robotic workflows that lack universal interoperability between hardware and software systems. Artificial intelligence models for anatomical recognition and fluorescence quantification are still early-stage prototypes, often trained on small datasets with limited external validation. Standardized benchmarks, large annotated video repositories, and transparent performance metrics are needed before regulatory approval and clinical deployment can be achieved.^62^

Finally, ethical and regulatory challenges accompany the integration of artificial intelligence and digital decision-support systems into surgical practice. Issues of data privacy, algorithmic transparency, and accountability in the event of diagnostic or procedural errors must be clearly defined. Equally important is preserving the surgeon's autonomy and ensuring that AI functions as an assistive—not substitutive—tool within the decision-making process. Despite the promising potential of digital and robotic technologies, their widespread implementation remains limited by substantial costs, need for dedicated infrastructure, and disparate access between high- and low-resource settings. Cost-effectiveness analyses are scarce and should become a research priority to ensure that the benefits of precision surgery can be translated into sustainable and equitable care.^75^

Additionally, the incorporation of artificial intelligence and advanced technologies into surgical decision-making raises important ethical considerations. Transparency of algorithmic training and decision-making, accountability in case of errors, and preservation of surgeon autonomy are essential to maintain trust and patient safety. Furthermore, the use of patient data for model training requires strict adherence to privacy and data governance standards. Therefore, ethical frameworks must evolve alongside technological innovation to guide responsible integration of AI into clinical practice.^76^

## Future perspectives

The pace at which new surgical milestones are achieved is now catalyzed by emerging technologies. In the early stages of innovation, the current lack of robust data demonstrating improved clinical outcomes from the incorporation of digital tools into surgical workflows must be interpreted in the context of their recent introduction. Maximum efforts should now be directed toward generating high-level evidence to substantiate the true benefits of digital surgery.^77^

In the near future, lymph node staging is expected to advance with optical biopsy technologies potentially enabling real-time diagnosis of isolated tumor cells, including newer iterations of FF-OCT specifically designed to optimize dynamic cell visualization.^78^ Future developments may also lead to the creation of robotic *drop-in* probes for in vivo optical analysis—currently unfeasible due to the mechanical stability required for image acquisition—and the use of deep learning models for autonomous diagnostic tissue assessment.^45^

In the field of intraoperative ultrasound, the development of high-frequency *drop-in* probes could overcome current limitations in detecting micrometastatic lymph nodes, adding the advantages of real-time, in vivo diagnostics. The application of radiomic algorithms could further enhance our ability to interpret subtle, yet unexplored, imaging features.^79^^,^^80^

Ongoing clinical trials exploring computer vision applications may soon yield promising results in differentiating benign from metastatic lymph nodes using laparoscopic and robotic video data.^62^ Further advancements are expected in image-based molecular staging, particularly in endometrial carcinomas. Tumor-specific molecular targets will enable the development of novel nuclear medicine protocols, improving the precision of sentinel node identification and enabling selective removal of only metastatic nodes—thus further minimizing the surgical burden of sentinel lymph node biopsy for staging purposes.^31^ Finally, the question of the future and the ultimate goal of research on future tools assessing the sentinel lymph node in vivo would be to omit to remove it if it is accurately considered as negative. Postsurgical lymphedema would then be *completely eradicated*. In the close future, the clinical relevance of these technologies should be measured not only by diagnostic accuracy or intraoperative efficiency, but also by their impact on patient-centered outcomes. Reductions in operative morbidity, lymphatic complications, and postoperative lymphedema are measurable benefits that translate into improved quality of life. Long-term data on recurrence and survival remain limited, and prospective trials should integrate these endpoints to demonstrate the true value of digital and image-guided surgery in oncologic care.

## Conclusion

The synergy between these technological innovations and the digital ecosystem is paving the way toward a new paradigm: the smart and hybrid operating room. In this environment, all these technologies will coexist in an integrated workflow—starting from preoperative image analysis to real-time intraoperative imaging via molecular image-guided surgery, and 3D reconstruction overlay through extended reality. This will not only enhance surgical precision but also serve as a powerful tool for surgical training and education.^81^

## Contributors

MP, DQ and BS conceptualized the study. All authors were involved in data curation, analysis and interpretation. MP, LL, DQ and BS drafted the manuscript with the input of all coauthors. All authors approved the final version. The corresponding author attests that all listed authors meet authorship criteria and that no other authors meeting the authorship criteria have been omitted.

## Declaration of interests

Barbara Seeliger declares that she is the recipient of a grant from the French National Agency for Research (Agence Nationale de la Recherche (ANR), 86 rue Regnault, 75,013 Paris, France) within the framework of the project AI-DIAL—Diagnostic Imaging of Adrenal Lesions (ANR-22-CE17-0019-01 and ANR-23-IACL-0004) and has a consultant agreement with Intuitive Surgical, both unrelated to the present study. Denis Querleu is a consultant for MIMARK Diagnostics S.L. (Barcelona, Spain). Matteo Pavone, Lise Lecointre, Nicolò Bizzarri, and Anna Fagotti have no conflict of interest to declare. The institutional funding did not affect any aspect of the conduction, analysis, or reporting of this study.
